# Combined Omega-3 Fatty Acid and Folic Acid Supplementation Reduces Neonatal Hypoxic-Ischemic Brain Injury via Anti-inflammatory and Anti-apoptotic Mechanisms

**DOI:** 10.5812/ijpr-163943

**Published:** 2025-10-24

**Authors:** Wendong Sun, Tanu Ojha, Vinod Kumar Verma, Siddhartha Kumar Mishra

**Affiliations:** 1 Department of Neurosurgery, Baoding First Central Hospital, Baoding, Hebei Province, 071000, China; 2 Department of Life Sciences and Biotechnology, Chhatrapati Shahu Ji Maharaj University, Kanpur – 228024 (U.P.), India; 3 Department of Biochemistry, University of Lucknow, Lucknow – 226007 (U.P.), India

**Keywords:** Hypoxia-Ischemia, Omega-3 Polyunsaturated Fatty Acid, Folic Acid, Apoptosis, Anti-inflammation

## Abstract

**Background:**

Neonatal hypoxic ischemia (HI) injury results in neuronal cell death, which remains clinically challenging to mitigate. Omega-3 polyunsaturated fatty acids (PUFAs) are known for their antioxidative and anti-inflammatory effects. Folic acid (FA) correlates with apoptosis in neural stem cells and neurons.

**Objectives:**

This study aimed to evaluate whether combined PUFA and FA supplementation mitigates neonatal HI brain injury by reducing apoptosis, inflammation, neurotransmitter imbalance, and electrophysiological dysfunction, thereby offering enhanced neuroprotection and functional recovery.

**Methods:**

Brain tissue damage, orthodromic population spike (OPS), and hypoxic injury potential (HIP) were measured. Amino acid neurotransmitter concentration in the hippocampus sections was measured. Markers of inflammation and apoptosis were assayed from HI-induced rat brains and lipopolysaccharide (LPS)-induced microglia BV-2 cells.

**Results:**

The HI caused severe damage to brain tissues that were potentially prevented by PUFA-FA by reducing the infarct size by 88%. The PUFA-FA treatment decreased the latency time (51 and 43 s) and increased swimming velocity (152 and 170 mm/s) on training days 3 and 5. The PUFA-FA showed an improved OPS decay time of 327 s, OPS recovery rate (62 s), and recovery amplitude (58 s). Whereas it caused an average 57% HIP incidence with a notably delayed onset (564 s) and duration (182 s). The PUFA-FA treatment also decreased the HI-induced release of amino acid neurotransmitters (Asp, Glu, and Gly) and GABA. The PUFA-FA suppressed the levels of proinflammatory cytokines and chemokines (iNOS, COX-2, TNF-α, IL-1β, and IL-6) and might mediate the inhibition of the NF-κB signaling pathway. The PUFA-FA reduced apoptosis as evidenced by lowered expression of AIF, caspase-3, and PARP genes.

**Conclusions:**

The PUFA and FA reduced HI-induced brain infarct size, with the combination showing greater protection compared to individual effects. Both improved cognitive performances, decreasing latency times and enhancing swimming velocity. The PUFA-FA supplementation synergistically restored memory, learning, and motor functions, highlighting strong neuroprotective effects against HI-induced neuronal degeneration and cognitive impairments.

## 1. Background

Neonatal hypoxic-ischemic brain injury (HIBI) is a major cause of neonatal morbidity and mortality, leading to severe neurological deficits, including cerebral palsy, developmental delays, and epilepsy (1). Numerous pathological disorders are brought on by hypoxic brain damage, yet the mechanism causing such hypoxic brain damage remains unknown. Polyunsaturated fatty acids (PUFAs) are necessary for the proper development of the brain, visual system, and immune system. The two primary n-3 PUFAs in the central nervous system are docosahexaenoic acid (DHA) and eicosapentaenoic acid (EPA) (2). The DHA and EPA are continuously used for the biogenesis and maintenance of neuronal membranes and are associated with many physiological functions, such as learning and memory (3). ω-3-PUFAs are extremely prone to peroxidation, and due to PUFA's bioavailability, the brain is especially susceptible to lipid peroxidation caused by reactive oxygen species (ROS) (4). In rodent models of HIBI, ω-3 PUFAs, particularly DHA, have been extensively researched (5, 6). Omega-3 PUFAs (especially DHA and EPA) confer robust neuroprotection in neonatal hypoxic ischemia (HI) by reducing infarct size, preserving BBB integrity, attenuating inflammation through microglial NF-κB inhibition, and activating the prosurvival PI3K/Akt pathway that ultimately enhances long-term cognitive and sensorimotor outcomes (4, 7). Bioactive DHA mediators (e.g., neuroprotectin D1) also confer rapid, mitochondria-linked protection in ischemia. Formulation nuances containing omega-3 emulsions and delivery forms can enhance infarct reduction and neuroprotection (8). Folic acid (FA), a synthetic form of folate (vitamin B9), is particularly significant for neuroprotection and brain function due to its involvement in the synthesis of neurotransmitters (9). The FA also exhibits antioxidant properties, protecting neurons from oxidative stress, a key factor in neurodegeneration. By supporting methylation and antioxidative defense mechanisms, folate helps preserve cellular integrity and function (10). The FA counters glutamate-induced excitotoxicity by activating the PI3K pathway, inhibiting GSK-3β, upregulating β-catenin, and reducing iNOS expression (11). It also attenuates ferroptosis by enhancing the SLC7A11/GSH/GPX4 antioxidant axis, increasing cystine uptake, and reducing lipid oxidative damage (12). Concurrently, FA mitigates HI-induced oxidative brain damage by activating the Nrf2/HO-1 pathway, elevating antioxidative defenses (GSH-Px, SOD), reducing ROS, lipid peroxidation, and cerebral edema, and inhibiting ferroptosis via upregulation of SLC7A11 and GPX4 (13). This study integrates behavioral, electrophysiological, histological, and molecular analyses, providing comprehensive evidence for the neuroprotective effects of PUFA-FA in neonatal HI. It demonstrates synergistic effects on cognition, synaptic function, inflammation, and apoptosis, linking mechanisms from hippocampal neurons to microglial responses. The use of both in vivo and in vitro models strengthens translational value. While findings are limited to rodent models, they may be extended to human studies. Long-term safety and dose optimization of PUFA-FA may further require assessment of the clinical applicability and therapeutic relevance.

## 2. Objectives

Combined, omega-3 and FA may target oxidative stress, inflammation, and apoptosis, underscoring strong multimodal neuroprotection in neonatal HI. This work aimed to explain the processes behind PUFA-assisted protection together with FA supplementation against ischemic neuronal injury using the HI injury neonatal mouse model and microglial cells. To the best of our knowledge, the effects of the combination of PUFA and FA as therapeutic interventions have not yet been documented.

## 3. Methods

### 3.1. Neuronal Hypoxia-Ischemia Model

Animal experiments were performed with the approval of protocols by the Institutional Animal Ethical Committee [Baoding First Central Hospital Approval No. LL2024(BD18)]. Scheduled pregnant female SD rats were randomly divided into four groups, each containing ten pregnant rats (n = 10). A sample size of ten animals per group ensures adequate statistical power, minimizes biological variability, and provides reliable comparisons between control and treatment groups while adhering to ethical standards of animal research. Group I (control group) was provided a standard diet. Group II (PUFA group) was provided with PUFA (DHA and EPA each 10 mg/g diet) (14). Group III (FA group) was provided with FA (10 mg/g diet) (15). Group IV (PUFA-FA group) was provided with PUFA and FA (as in groups II and III). Post-delivery of pregnant rats, seven-day-old rat pups (postnatal day 7, P7) were exposed to HI brain injury. Rats were anesthetized for performing ligation of the left common carotid artery. Then, rat pups were given a 1.5-hour recovery period and then placed into a chamber with a humidified atmosphere of 8% O_2_/92% N_2_ for 2.5 hours.

### 3.2. Assessment of Brain Damage

The cresyl violet staining method was used to stain brain sections (n = 3) to evaluate neuronal damage by calculating the quantity of surviving tissue per segment and determining the volume loss in the ipsilateral versus contralateral hemisphere.

### 3.3. Behavioral Assessment of Neurocognitive Functions

The Morris Water Maze (MWM) test was applied for the assessment of spatial learning and memory as functions of behavioral alterations (16). Rats (n = 5) of day P28 were recruited for the MWM test on a black-coated pool (dimension 100 cm × 50 cm) filled with water. A camera was placed 2.5 m above the pool to record the activities. Latency time was measured, which represents the spatial learning ability. Swimming velocity was also measured as one of the parameters. Animals were given the MWM exercise to test their spatial memory. The Reference Memory procedure, which included five training days with four trials per day and a 20-minute inter-trial interval, was applied to the animals. On the sixth day, a probe experiment was conducted. Animals from each quadrant were randomly assigned to the water in each training trial. Rats were lowered through the water and left on the platform for ten seconds if they did not make it to the platform. The platform was removed during the probing experiment, and the following variables were noted: The duration spent in the target and opposite quadrants, the number of crossings in the platform region, and the latency to reach the platform area. Probe trials were performed after each day of training to determine the animals’ actual learned ability by identifying the platform area as a mark of spatial memory ability. For this experiment, the platform was removed from the pool, and rats were allowed free swimming during a 100-second time period, and then the time spent in each quadrant was recorded.

### 3.4. Recording of Extracellular Potential

For electrophysiology, the CA1 and CA3 hippocampal regions are selected because they form a classic neural circuit involved in learning and memory. The CA3 auto-associative network projects to the CA1 region via Schaffer collaterals, making it ideal for studying synaptic plasticity, such as long-term potentiation (17). The extracellular potential was recorded after electric probes were placed into the Schaffer lateral fiber at CA3 of rats (n = 5). Ten-second intervals were used to provide the stimuli (0.6 mA, 0.1 Hz, and 100 ms). At CA1, evoked potentials such as hypoxia injury potential (HIP) and orthodromic population spike (OPS) were measured. For the experiments, only brain slices with consistent OPS (43 mV) for at least 20 minutes were chosen. Following a 15-minute incubation period in ASCF without glucose and oxygen (ASCFOGD), hypoxic groups showed normal ASCF perfusion. Every OPS in the treatment and hypoxic groups was noted prior to its removal. The proportion of hippocampus slices in which HIP was seen following oxygen deprivation is known as the HIP incidence.

### 3.5. HPLC Analysis of Brain Tissues

Rat brain hippocampus slices (n = 3) were incubated for 2 hours at 37°C in ASCF with and without glucose (10 mM). Each group's ASCF after 15 minutes of perfusion was centrifuged, and the clear supernatant was subjected to HPLC analysis. Sodium acetate (0.5 M, pH 6.0) with 0.05% THF was used as mobile phase A and methanol as mobile phase B at a rate of 0.9 mL/min at an emission wavelength of 330 nm and an excitation wavelength of 456 nm. The concentrations of Asp, Glu, Gln, Gly, and GABA in mM in perfusion ASCF were calculated.

### 3.6. Microglial Cell Cultures and Treatments

The murine microglia cells BV-2 (2 × 10^5^ cells/ml) were plated onto 24-well plates and cultured in DMEM media supplemented with 10% FBS, penicillin (100 units/mL), streptomycin (100 μg/mL), and glutamine (2 mM). Cells were incubated for 24 hours at 37°C in a 5% CO_2 _humidified atmosphere in a CO_2_ incubator. The PUFA was dissolved in DMSO and then diluted with culture media to a final concentration of DMSO 0.05%, which served as a vehicle control, while FA was prepared in culture media. Cells were treated with PUFA and FA for 6 hours and then stimulated with 1 μg/mL of lipopolysaccharide (LPS). Each experiment was replicated a minimum of three times.

### 3.7. Assessment of Inflammatory Markers

The cytokine release was measured using the cell supernatant from cultured BV-2 cells. The Griess reagent was used to assay the formation level of NO, and ELISA assay kits were used to measure the levels of IL-1β, IL-10, and TNF-α. The experiment was replicated in triplicate.

### 3.8. Gene Expression Analysis by qPCR

Total RNA was extracted from hippocampal sliced tissues (n = 3) using the Trizol reagent, followed by conversion to cDNA using a Reverse Transcriptase kit. Then, quantitative amplification of target genes was performed using gene-specific primers (Appendix 2 in Supplementary File) on an Applied Biosystems 7500 real-time PCR System using the SYBR Green detection system. For normalization by the ΔΔCt method, Ct values of target genes were normalized to those of 18S. Then, ΔCt of treated and control samples was compared (ΔΔCt) for the calculation of relative expression, giving fold-change in gene expression.

### 3.9. Statistical Analysis

Data were expressed as mean ± standard deviations (SD) for a minimum of three experimental repeats. For behavioral experiments, One-Way ANOVA followed by Tukey's post-hoc test was applied for multiple comparisons. The Student's paired *t*-test was applied for cell-based and molecular assays. Statistical significance was defined as P-values less than 0.05.

## 4. Results

### 4.1. Polyunsaturated Fatty Acid and Folic Acid Exert Long-Term Neuroprotective Effects

**Figure 1.**
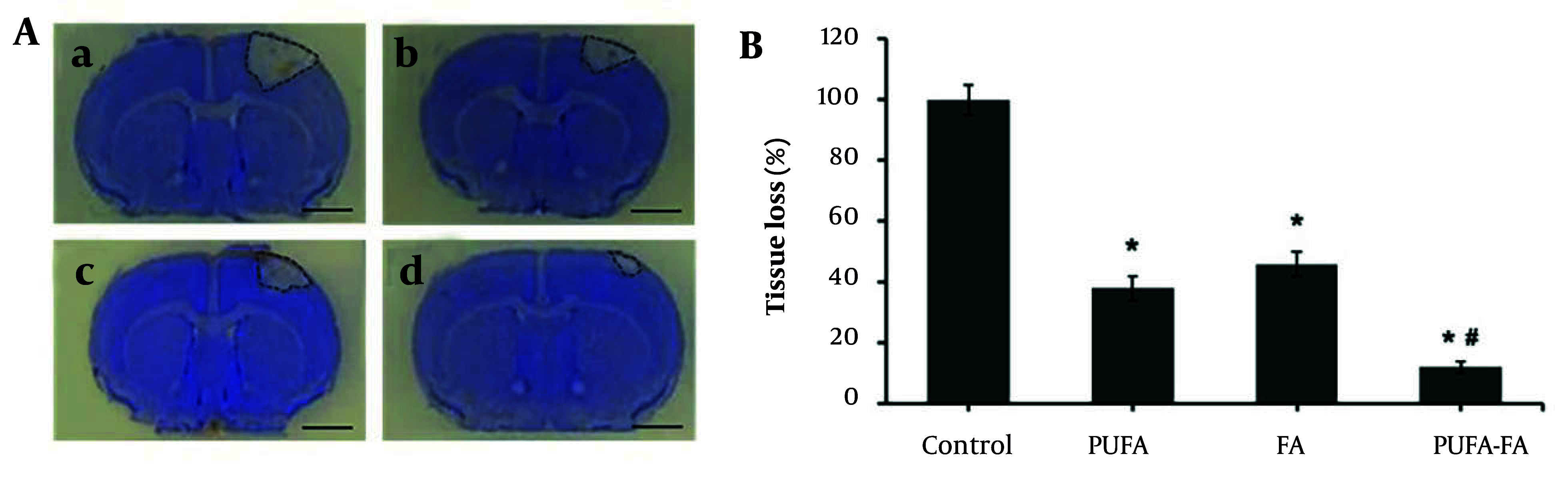
Effect of polyunsaturated fatty acid (PUFA) and folic acid (FA) on long-term neuroprotection against neonatal hypoxic ischemia (HI) brain injury. A, representative photographs of cresyl violet-stained brain coronal sections (n = 3): a, HI control group; b, PUFA-treated group; c, FA-treated group; d, PUFA-FA-treated group. B, quantification of tissue loss and representation of data as a percentage of HI control (100%; * P < 0.05 vs. control; # P < 0.05 vs. PUFA or FA groups; injury areas are marked with lines across them; scale bar 1 mm).

The physiological marker of brain degeneration was assessed in the form of an infarct area after the HI insult (Figure 1A). The HI-induced brain tissues showed a loss in the cortex, striatum, and hippocampus, and a sizable region of tissue loss as shrunken and dying cells. These changes were restored by PUFA and PUFA-FA fed mice groups. Compared to the HI-induced brain infarct (set as 100%), the overall tissue loss decreased to 38 ± 4% and 46 ± 4% as a result of PUFA and FA treatments, respectively (Figure 1B). The comparison of infarct size between PUFA (38 ± 4%) and FA (46 ± 4%) groups showed a statistically significant increase with P-values < 0.05. In contrast, the combined treatment with PUFA and FA showed a notable reduction in infarct size (12 ± 2%) with P-values < 0.01.

**Figure 2.**
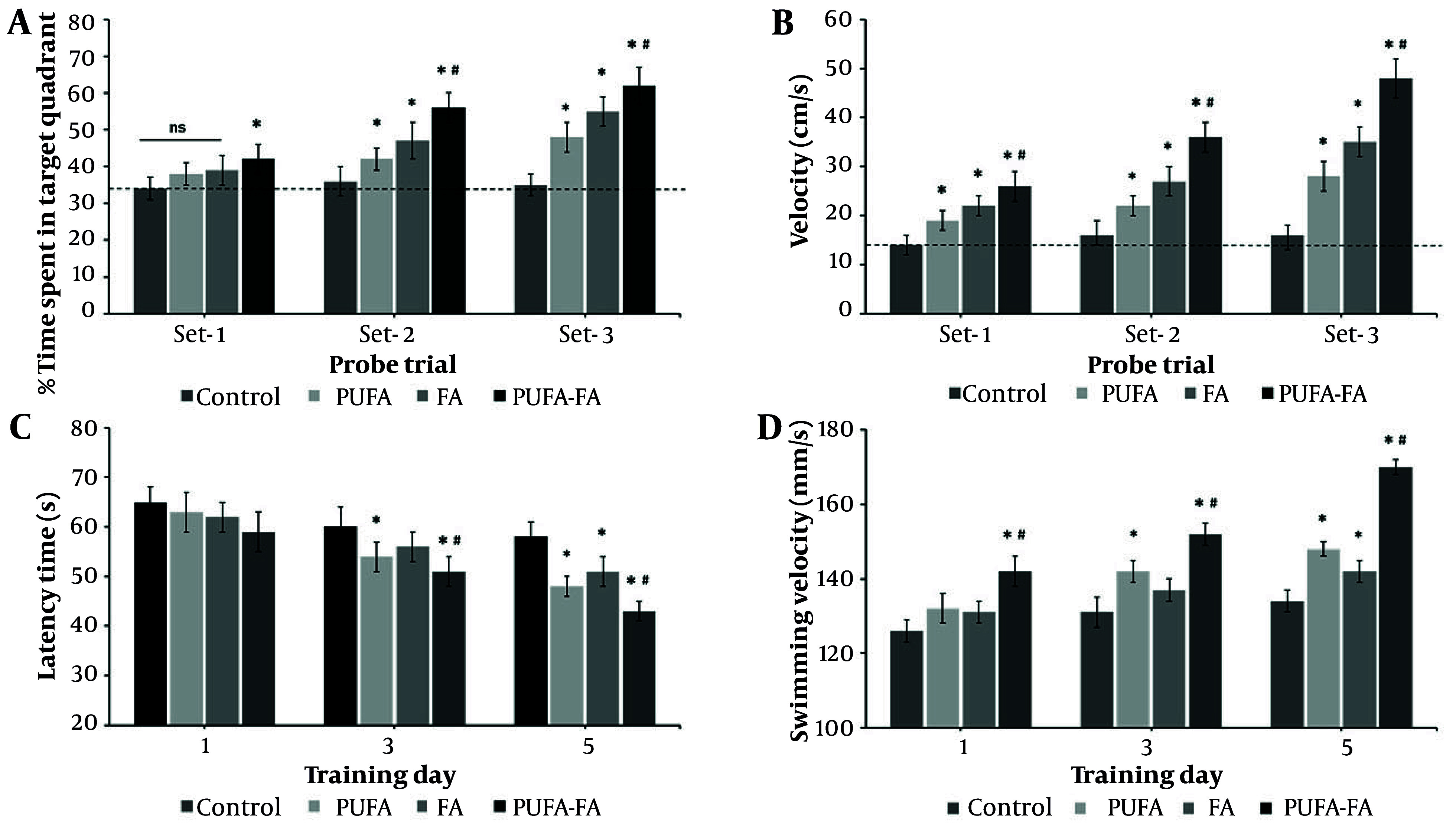
Effect of polyunsaturated fatty acid (PUFA) and folic acid (FA) on hypoxic ischemia (HI)-induced learning behavior: A, probe trial performance after training days in the Morris Water Maze (MWM) test and assessment of the percentage of time spent in the target quadrant; B, assessment of swimming speed during probe trials test. The dotted line represents the level in control that remains unchanged in all the probe trials; C, MWM test to measure the latency time (s) to find the hidden platform from training days 1 to 5; D, the swimming velocity (s) of rats was measured from training day 1 to 5 (n = 5 per group; * P < 0.05 vs. control; # P < 0.05 vs. PUFA or FA groups).

The MWM probe trial is a behavioral test used to assess spatial learning and memory in animals, where a key measure is the animal's tendency to spend more time or swim a greater distance in the quadrant where the escape platform was previously located. This data is commonly presented as percentages of time or distance in the target quadrant, reflecting memory retrieval and cognitive function by comparing the performance of different groups. The probe trial performed in the MWM test showed no preference for the target quadrant during the first probe trial (compared to control with a dotted line; Figure 2A). During the second probe trial, all groups showed significantly more time in the target quadrant, which increased even more in the PUFA-FA combination group (P < 0.01). Likewise, animals with HI showed slower swimming speed during all probe trials (Figure 2B), which was notably increased in all the treatment groups, especially the PUFA-FA group (P < 0.01). Latency time was measured to assess the neurocognitive abilities of rats following HI exposure and PUFA-FA supplementation (Figure 2C). The control group rats' latency times (s) were 65 ± 3, 60 ± 4, and 58 ± 3 on training days 1, 3, and 5. Latency times on different training days within the control group remained statistically not significant (Figure 2C). In PUFA-treated rats, latency time decreased to 54 ± 3 and 48 ± 2 s on training days 2 and 5. It was reduced to some extent in the FA-treated group (51 ± 3 s) on training day 3. In the PUFA-FA combination treatment group, latency time decreased to 51 ± 3 and 43 ± 2 s on training days 2 and 5.

The swimming velocity test demonstrates that rats in the control group showed a swimming velocity of 126 ± 4 to 134 ± 6 mm/s on training days 1 to 5 (Figure 2D). The PUFA-treated rats showed an increased swimming velocity of 142 ± 3 and 148 ± 5 mm/s on training days 2 and 5, while it was increased to 142 ± 3 mm/s on training day 3 in the FA-treated group. Rats treated with a combination of PUFA-FA showed synergistically increased swimming velocity (142 ± 3, 152 ± 3, and 170 ± 6 mm/s on training days 1, 3, and 5, respectively). Notably, PUFA-FA supplementation helped to restore cognitive problems and enhanced adult learning and memory abilities. These results also suggest that supplementing with PUFA-FA improved the memory loss and cognitive impairment caused by HI, with improvements in swimming velocity.

### 4.2. Polyunsaturated Fatty Acid and Folic Acid Treatment on Orthodromic Population Spike and Hypoxic Injury Potential After Neonatal Ischemia Hypoxia

**Figure 3.**
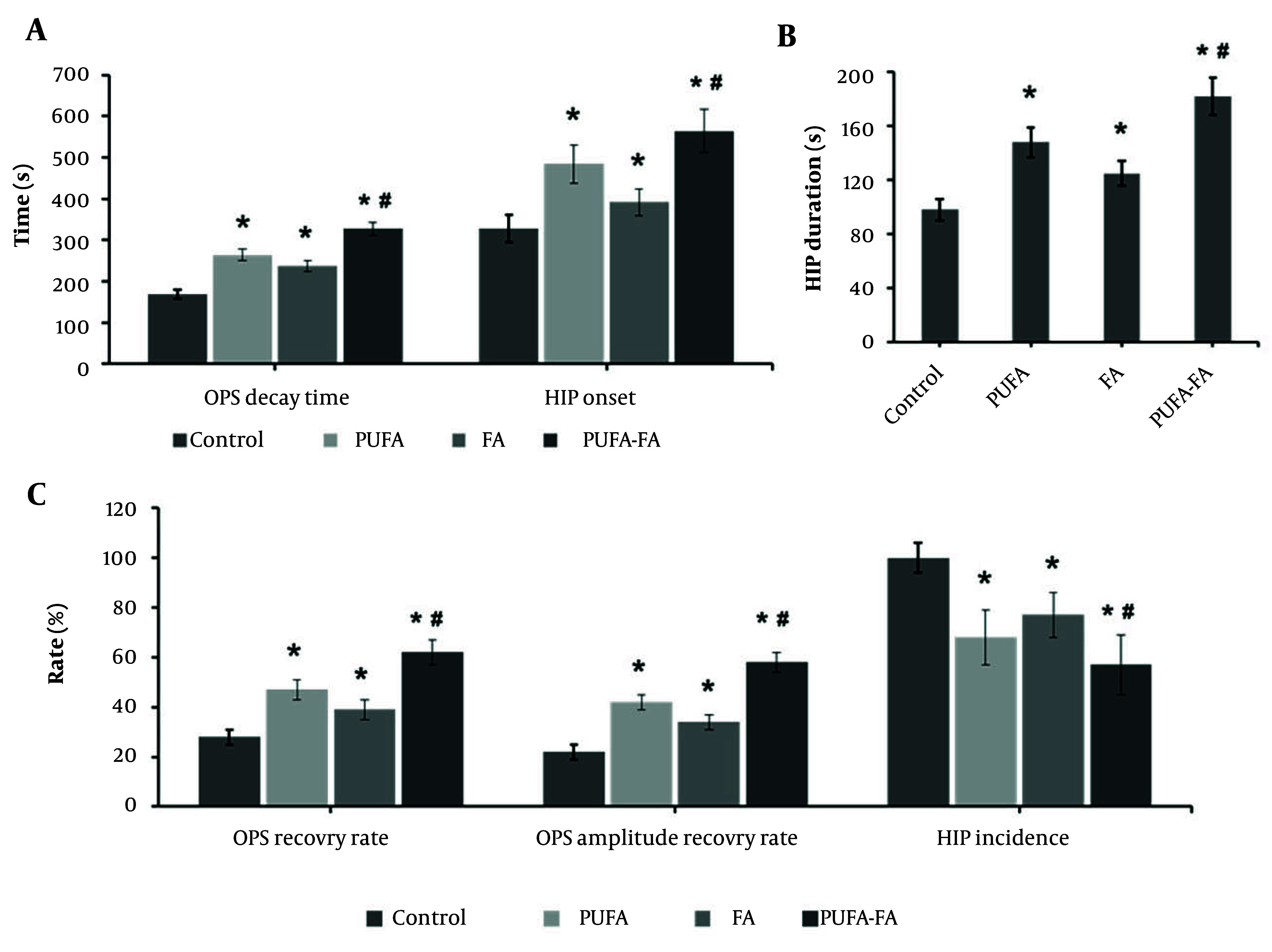
Effect of polyunsaturated fatty acid (PUFA) and folic acid (FA) on hypoxic ischemia (HI)-induced orthodromic population spike (OPS) and hypoxic injury potential (HIP): A, OPS decay time (s) and HIP onset (s); B, HIP duration (s); C, OPS recovery rate (%), OPS amplitude recovery (%), and HIP incidence (%; n = 5 per group; * P < 0.05 vs. control; # P < 0.05 vs. PUFA or FA groups).

One of the ways to assess neuronal tolerance is the OPS decay, as neuron damage will be delayed if the beginning of HIP is delayed or prolonged. At the CA1 region, OPS and HIP were measured (Figure 3). The average OPS decay time (Figure 3A) in the control group was 168 ± 11 s, with an OPS recovery ratio of 28% (Figure 3C) and an amplitude of recovery of 22% (Figure 3C). The OPS decay time was considerably longer in the PUFA-treated group (264 ± 14 s) with a higher OPS recovery rate (47 ± 4 s) and recovery amplitude (42 ± 3 s). The FA-treated group showed an OPS decay time of 238 ± 13 s, an OPS recovery rate of 39 ± 4 s, and a recovery amplitude of 34 ± 3 s. In contrast, the combination of PUFA-FA showed an improved ratio of these parameters with an OPS decay time of 327 ± 16 s, OPS recovery rate of 62 ± 5 s, and recovery amplitude of 58 ± 4 s.

The incidence of HIP in the control group was approximately 100%, with an average onset time of 328 ± 34 s and an average duration of 98 ± 8 s (Figures 3A - C). The PUFA-treated group showed a decrease in the incidence of HIP to 68%, with a delayed onset of HIP (484 ± 46 s) and an increased duration of HIP (148 ± 11 s). The FA-treated group showed a 77% incidence of HIP with a delayed onset of HIP (392 ± 32 s) and an increased duration of HIP (125 ± 9 s). Whereas the PUFA-FA combination-treated group showed an average 57% HIP incidence with a notably delayed onset (564 ± 52 s) and duration (182 ± 14 s). Delayed OPS decay indicates preserved synaptic transmission and membrane excitability in CA1, reflecting better energy homeostasis and Na^+^/K^+^-ATPase function during HI. A late HIP onset signifies postponement of ischemic/spreading depolarization, lowering ionic collapse, glutamate-driven excitotoxicity, and ATP depletion, which is a key determinant of irreversible injury. Together, these delays suggest a larger salvageable penumbra, enhanced neuroprotection, and improved likelihood of functional recovery (18). These results show that synaptic communication between hippocampus neurons was damaged by hypoxia and that the recovery ratio and amplitude of OPS were increased by PUFA and FA supplementation.

### 4.3. Polyunsaturated Fatty Acid and Folic Acid Regulate Release of Amino Acid Neurotransmitters

HPLC analysis was used to assess the amount of amino acid neurotransmitters from hippocampus tissues and presented as mean ± SD in Table 1. The HI control group's neurotransmitter concentrations of GABA, Asp, Glu, Gln, and Gly were increased to the levels of 2.85, 2.65, 2.60, 1.25, and 2.15 pmol, respectively. In the PUFA-treated group, the levels of GABA, Asp, Glu, and Gly were reduced to 2.15, 2.20, 2.10, and 1.85 pmol, respectively. The FA-treated group showed some decrease in the levels of GABA, Asp, Glu, and Gly to 2.45, 2.40, 2.35, and 1.95 pmol, respectively. In contrast, the combination of PUFA-FA showed a notable reduction in the levels of GABA, Asp, Glu, and Gly to 1.90, 2.05, 1.90, and 0.65 pmol, respectively. These observations indicate that EAA neurotransmitters were elevated due to HI injury in the brain, which was controlled by PUFA and FA supplementation.

**Table 1.** The Effect of Polyunsaturated Fatty Acid and Folic Acid on the Release of Neurotransmitters and Neuronal Amino Acids from the Hippocampus ^a^

Groups	GABA	Asp	Glu	Gln	Gly
**Control**	2.85 ± 0.20	2.65 ± 0.20	2.60 ± 0.15	1.25 ± 0.10	2.15 ± 0.20
**PUFA**	2.15 ± 0.15 ^b^	2.20 ± 0.10 ^b^	2.10 ± 0.10 ^b^	1.10 ± 0.10	1.85 ± 0.20 ^b^
**FA**	2.45 ± 0.20 ^b^	2.40 ± 0.20 ^b^	2.35 ± 0.20 ^b^	1.15 ± 0.10	1.95 ± 0.20
**PUFA/FA**	1.90 ± 0.20 ^b, c^	2.05 ± 0.10 ^b, c^	1.90 ± 0.15 ^b, c^	1.05 ± 0.10	1.65 ± 0.10 ^b, c^

Abbreviations: PUFA, polyunsaturated fatty acid; FA, folic acid.

^a^ Values are recorded in pmol/L and expressed as mean ± standard deviations (SD).

^b^ P < 0.05 vs. control.

^c^ P < 0.05 vs. PUFA or FA.

### 4.4. Polyunsaturated Fatty Acid and Folic Acid Suppress Microglial Inflammation

**Figure 4.**
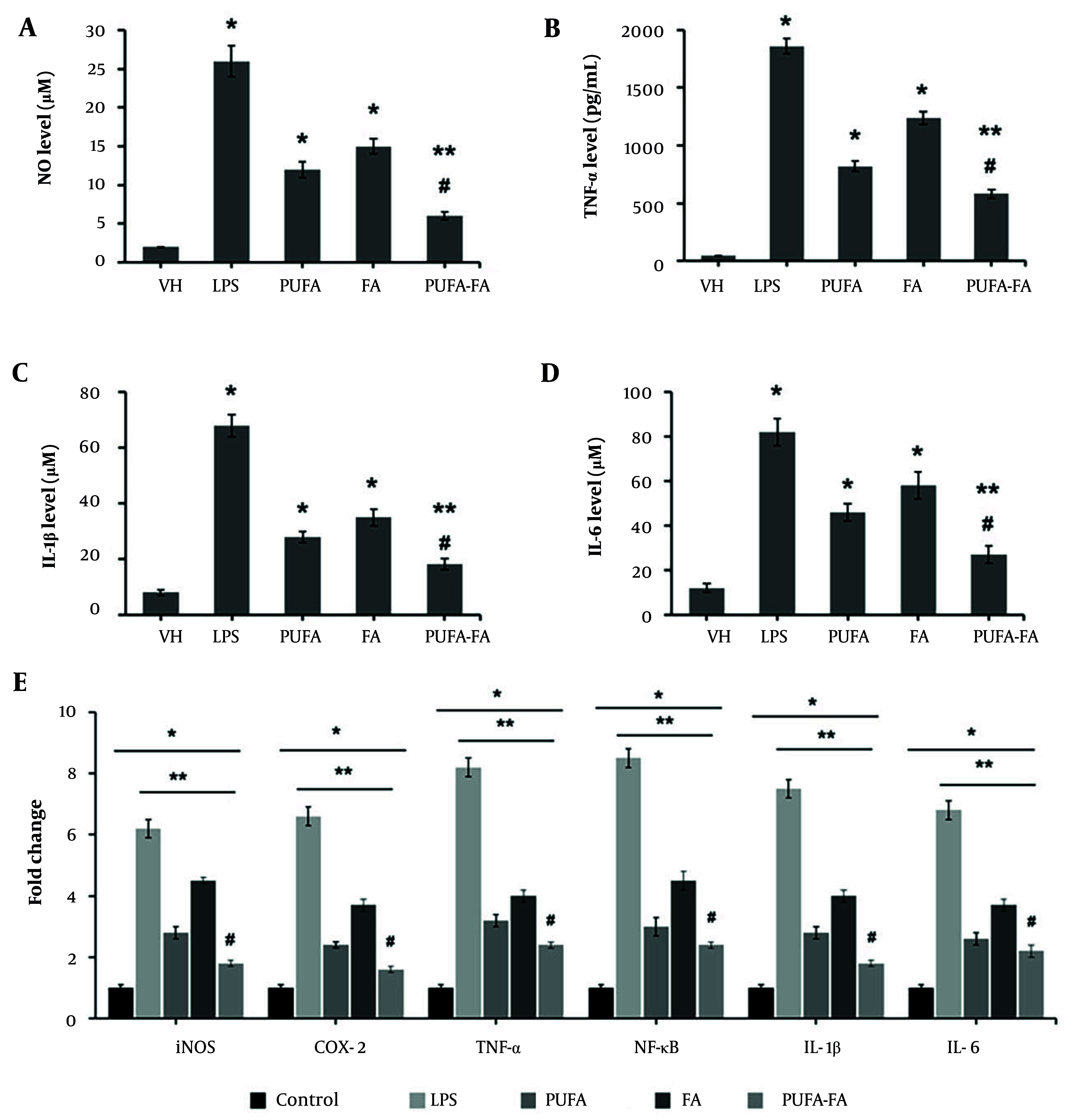
Effects of polyunsaturated fatty acid (PUFA) and folic acid (FA) on lipopolysaccharide (LPS)-induced inflammatory markers in BV-2 cells: ELISA-based analysis of inflammatory markers was performed in triplicate (n = 3) for (A) nitric oxide (μM), (B) TNF-α (pg/mL), (C) IL-1β (μM), (D) IL-6 (μM), and (E) qPCR for assessment of gene expression for iNOS, COX-2, TNF-α, NF-κB, IL-1β, and IL-6 (* P < 0.05 vs. control; ** P < 0.05 vs. LPS; # P < 0.05 vs. PUFA or FA groups).

Microglia, which are resident macrophages in the brain, play a crucial role in the development and spread of the inflammatory response as well as the generation of cytokines in newborn HI brain injury (19). The results obtained from the ELISA-based assay were computed (Table 2). The level of NO was elevated to 26 ± 2 μM in LPS (1 μg/mL) treated cells, while it was nominal in vehicle-control cells (2 ± 0.1 μM; Figure 4). Treatment of cells with PUFA (20 μM) and FA (10 μg/mL) caused a reduction in the NO level to 12 ± 1 and 15 ± 1 μM, respectively. In contrast, the combination of PUFA-FA caused a dramatic decrease in the NO level (6 ± 0.5 μM) (Figure 4A). 

**Table 2.** Effect of Polyunsaturated Fatty Acid and Folic Acid on the Concentration of Nitric Oxide and Cytokines ^a, b^

Groups	NO		TNF-α		IL-1β		IL-6	
	Conc. (μM)	% (To Control)	Conc. (pg/mL)	% (To Control)	Conc. (μM)	% (To Control)	Conc. (μM)	% (To Control)
**Control**	(2 ± 0.10)	100	(45 ± 2.0)	100	(8 ± 1.0)	100	(12 ± 2.0)	100
**LPS**	(26 ± 2.0) ^c^	1300	(1860 ± 65) ^c^	413	(68 ± 4.0) ^c^	850	(82 ± 6.5) ^c^	683
**PUFA**	(12 ± 1.0) ^c, d^	600	(820 ± 45) ^c, d^	182	(28 ± 2.0) ^c, d^	350	(46 ± 4.5) ^c, d^	383
**FA**	(15 ± 1.0) ^c, d^	750	(1240 ± 55) ^c, d^	276	(35 ± 3.0) ^c, d^	436	(58 ± 6.0) ^c, d^	483
**PUFA-FA**	(6 ± 0.5) ^c, d, e^	300	(580 ± 40) ^c, d, e^	129	(18 ± 2.0) ^c, d, e^	225	(27 ± 4.0) ^c, d, e^	225

Abbreviations: LPS, lipopolysaccharide; PUFA, polyunsaturated fatty acid; FA, folic acid.

^a^ Values are expressed as mean ± standard deviations (SD).

^b^ N = 3 per group.

^c^ P < 0.05 vs. control.

^d^ P < 0.05 vs. LPS.

^e^ P < 0.05 vs. PUFA or FA.

The level of TNF-α was increased multifold in LPS-treated BV-2 cells (1860 ± 65 pg/mL) compared to control (45 ± 2 pg/mL), which was suppressed by PUFA and FA treatment to 820 ± 45 and 1240 ± 55 pg/mL individually, and by their combination to 580 ± 40 pg/mL (Figure 4B). The level of IL-1β in control BV-2 cells was 8 ± 1 μM, which rose to 68 ± 4 μM when exposed to LPS. This level was suppressed by PUFA and FA treatment to 28 ± 2 and 35 ± 3 μM, and their combination reduced it to 18 ± 2 μM (Figure 4C). Similarly, the level of IL-6 in control BV-2 cells was 12 ± 2 μM, which rose to 82 ± 6 μM when exposed to LPS, and it was suppressed by PUFA and FA treatment to 46 ± 4 and 58 ± 6 μM, with their combination reducing it to 27 ± 4 μM (Figure 4D). 

The qPCR results (Figure 4E) show that the gene expression for iNOS, COX-2, TNF-α, NF-κB, IL-1β, and IL-6 was elevated to 6 to 8.5-fold when treated with LPS (1 μg/mL) compared to the control (one-fold). The level of expression of these genes was reduced to 2.4 to 3.2-fold by treatment with PUFA (20 μM), while it was 3.7 to 4.5-fold in FA-treated BV-2 cells. In contrast, the combination of PUFA-FA caused a notable reduction in the expression of these genes to 1.6 to 2.4-fold. These results show the anti-inflammatory potentials of PUFA, which were further enhanced by FA.

### 4.5. Polyunsaturated Fatty Acid and Folic Acid Inhibited Inflammation and Apoptosis in Hypoxic Ischemia (HI)-Exposed Brain

**Figure 5.**
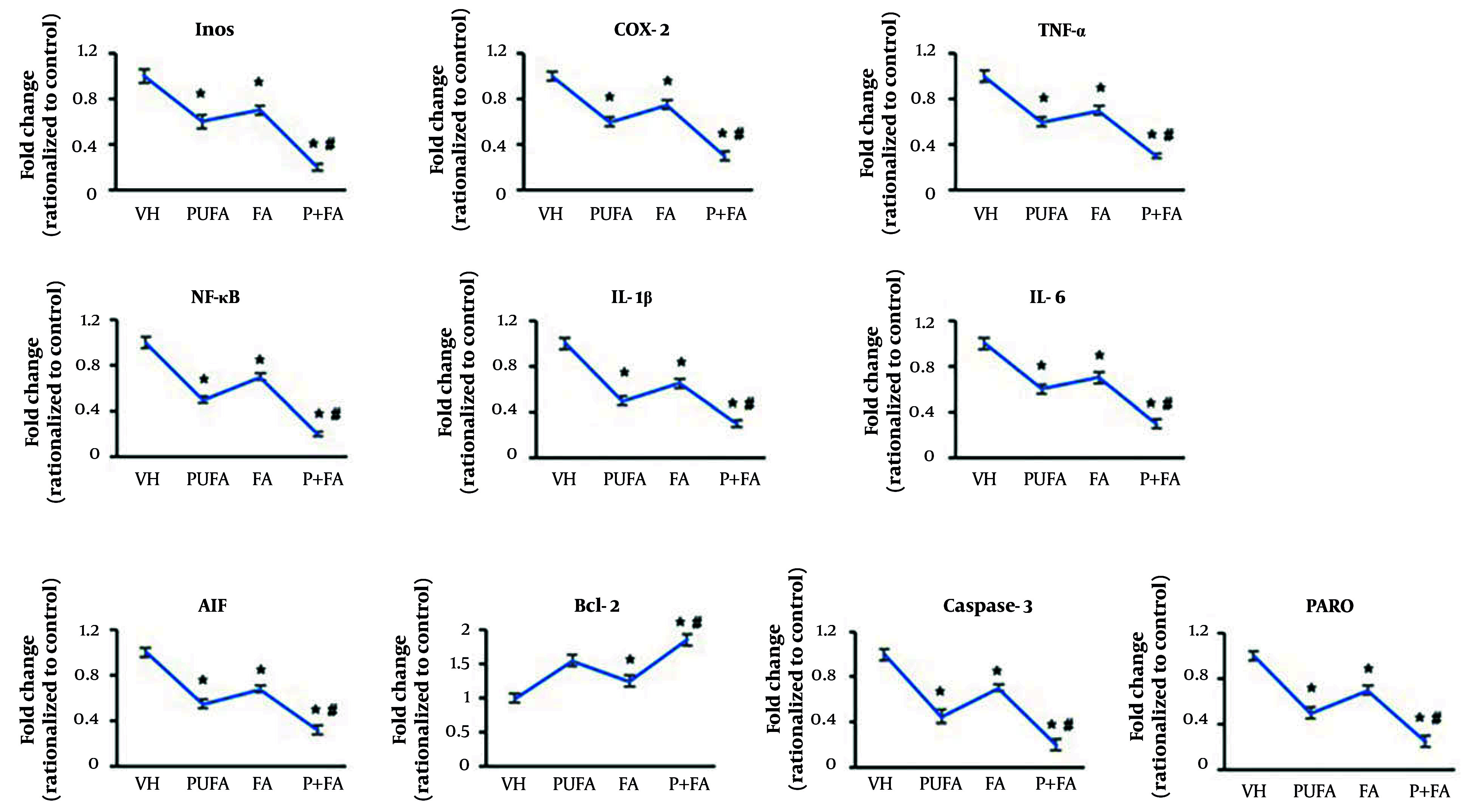
Effects of polyunsaturated fatty acid (PUFA) and folic acid (FA) on hypoxic ischemia (HI)-induced inflammation and apoptosis. A, qPCR for assessment of expression of inflammation-related genes: iNOS, COX-2, TNF-α, NF-κB, IL-1β, and IL-6; B, qPCR for assessment of expression of apoptosis-related genes: AIF, caspase-9, caspase-3, and PARP (performed in triplicate; n = 3; * P < 0.05 vs. control; # P < 0.05 vs. PUFA or FA groups).

Next, we assessed the expression of inflammation and apoptosis-related genes in brain tissues by qPCR (Figure 5A). Normalized to HI control (one-fold), treatment with PUFA reduced the expression of NF-κB to 0.5-fold. The expression levels of iNOS, COX-2, TNF-α, and IL-16β were reduced to 0.6-fold, and IL-1β to 0.5-fold. The FA-treated groups showed 0.65 to 0.75-fold expression of these genes. In contrast, the combination of PUFA-FA dramatically reduced the expression of NF-κB and iNOS to 0.2-fold and other genes to 0.3-fold.

Compared to the HI control group (one-fold), the expression level of AIF was reduced to 0.55-fold by PUFA supplementation, possibly due to its activation mechanism (Figure 5B). The FA also reduced AIF’s expression level to 0.68-fold, which was dramatically decreased by the PUFA-FA combination (0.32-fold). The expression of Bcl-2 was increased to 1.55-fold by PUFA supplementation, while FA also increased the level of Bcl-2 by 1.25-fold, compared to the HI control group (one-fold). The combination of PUFA-FA led to a notable increase in the expression of Bcl-2 (1.85-fold).

The levels of caspase-3 and PARP were reduced to 0.45 and 0.5-fold, respectively, by PUFA supplementation. The FA also suppressed their expression levels to 0.7-fold. Moreover, the combination of PUFA-FA notably suppressed the expression of caspase-3 and PARP to 0.2 and 0.25-fold, respectively. The expression levels of NF-κB, caspase-9, caspase-3, PARP, and β-actin proteins were measured (Appendix 1 in Supplementary File). The exposure to PUFA and FA protects the rat brain from HI insult through the prevention of damage to synaptic processes and lessening the neuronal apoptotic loss.

## 5. Discussion

The HI has been linked to several pathophysiological variables, such as oxidative stress, excitotoxicity, and inflammatory mediators. This study explored the protective effects of PUFA and FA on HI-exposed neuronal degeneration in the neonatal rats' brains. A synergistic reduction in infarct size (12%) was observed with the combined PUFA and FA treatment, suggesting that FA and PUFA have a potent neuroprotective effect against neonatal HI injury. The MWM test assessed memory and cognition function, showing that on training days 2 and 5, the latency time dropped to 51 and 43 seconds in the PUFA-FA combination treatment group. The PUFA-FA combination showed a synergistic improvement in swimming velocity as a marker of neuromotor efficiency. The HI eventually resulted in neurocognitive deficits and effects on rats’ memory and learning, while the PUFA-FA supplementation remarkably improved these changes.

The most hypoxia-sensitive cells are the pyramidal neurons in the hippocampus, with the CA1 pyramidal neurons being particularly susceptible. Due to the formation of synapses between CA1 and CA3, when the Schaffer collateral occurs in CA3, the OPS can be observed in CA1 (20). The OPS amplitude correlates inversely with HIP. During hypoxia, OPS is suppressed, reflecting synaptic failure, while reoxygenation or hyperoxic stimuli can evoke oxygen-induced potentiation, marking transient synaptic hyperexcitability and plasticity (2). The times to OPS disappearance and the presence of HIP were markedly delayed, and the rate and amplitude of OPS recovery were remarkably increased, accompanied by a decreased presence of HIP. These results indicated that PUFA-FA could confer protective effects on the HI-induced injury of the brain. Establishing a relationship between electrophysiological improvements and behavioral outcomes is necessary to confirm functional recovery. Higher oscillatory activity and better synaptic responses in the hippocampus are often predictive of better performance on spatial memory tests, such as the MWM performance (16). Similarly, the restoration of motor-evoked potentials has been shown to predict improvements in locomotor coordination and motor function in preclinical experimental models of neurodegeneration (21).

Correlating improvements in OPS and HIP with standardized behavioral assays for cognition and motor function, this study demonstrates that electrophysiological changes represent significant functional restoration associated with PUFA-FA treatment. The HI brain injury process is largely mediated by EAAs, such as Glu and Asp (22, 23), which are key components in the CA1 area of the hippocampus that were restored by PUFA-FA supplementation. The PUFAs can differentially affect Glu and GABA metabolism and concentrations depending on factors like the pathophysiology of the brain. While some studies show n-3-PUFAs can alter neurotransmitter levels and influence uptake mechanisms, others find minimal effects on GABA or glutamate in certain contexts. For example, arachidonic acid (an n-6 PUFA) has been shown to inhibit glutamate uptake more strongly than GABA uptake in neurons and astrocytes (24). The FA indirectly influences amino acid neurotransmitters (Glu, Asp, GABA) by participating in one-carbon transfers, which are vital for the synthesis of nucleic acids and other molecules. It may increase the expression of the GABA(A)-B 1 receptor subunit and may also impact monoamine neurotransmitters, contributing to depression-like behaviors (25).

This study also explored the effects of PUFA-FA on LPS-stimulated microglial cells, which are essentially immune cells located in the CNS. The NO level was increased to about 25-fold in LPS (1 μg/mL) treated BV-2 cells, which was reduced to about 6-fold with PUFA-FA treatment. The levels of iNOS, COX-2, TNF-α, NF-κB, IL-1β, and IL-6 were similarly controlled by PUFA-FA treatment in LPS-induced BV-2 cells, suggesting the anti-inflammatory effects of PUFA. Intracellular proteins known as suppressors of cytokine signaling (SOCS) proteins prevent cytokine signaling in a broad range of cell types, including microglia that express SOCS proteins (26).

A notable observation is the regulation of the expression of NF-κB in LPS-induced BV-2 cells and HI-exposed rat brains, which emerged as a central molecule regulating the mechanism of action. The in vivo neonatal HI paradigm provided additional confirmation of the PUFA inhibitory effect on NF-κB activation. The DHA and EPA were shown to regulate the levels of NF-κB-p65 in microglial BV-2 cells stimulated with LPS by blocking the nuclear translocation of p65 (14). The PUFA-FA combination significantly reduced the expression of caspase-3 and PARP, confirming that apoptosis was stimulated in hippocampal regions of the hypoxic brain. Similarly, propofol was reported to increase the expression of the pro-apoptotic gene Bcl-xL, which initiated caspase-3 and PARP-mediated neuronal apoptosis.

By suppressing oxidative stress, neuroinflammation, and apoptosis, the brain's cortex and hippocampus showed improved learning memory impairments and neurodegenerative cognitive defects (27, 28). The PUFAs play crucial roles in neuronal membrane structure and function, influencing signal transmission and neurogenesis. While FA is essential for one-carbon metabolism, DNA synthesis, and methylation processes, including the neutralization of neurotoxic homocysteine. Together, PUFAs and FA exert synergistic neuroprotective effects by supporting brain health through maintaining cell membrane integrity, regulating gene expression, reducing inflammation, and promoting optimal DNA methylation and demethylation, thereby preventing neuronal damage and cognitive decline (29, 30).

### 5.1. Conclusions

This investigation shows that PUFA and FA supplementation improved long-term neurological outcomes following neonatal HI injury, decreased brain damage, and markedly decreased cerebral inflammation and apoptosis. PUFA and FA mitigated HI-induced brain damage by reducing infarct size, restoring neuronal survival, and enhancing synaptic function. Their combination synergistically suppressed oxidative stress, inflammation, and apoptosis, leading to improved memory, learning, and motor performance, thereby demonstrating potent neuroprotection through multimodal mechanisms against HI-induced neurodegeneration and cognitive impairments. It also demonstrates how PUFA and FA protect against HI by at least partially inhibiting the inflammatory response mediated by microglia. The PUFA and FA were found to have a strong protective effect against brain damage and functional deficits caused by HI.

Summarily, PUFA-FA prevented hypoxia-induced damage to synaptic processes, reduced neuronal loss, and shielded the rat brain against hypoxic ischemic injury through a plausible mechanism of regulating membrane fluidity and integrity, reducing DNA damage and apoptosis, quenching free radicals’ generation and ROS levels, and suppressing inflammatory cytokines and chemokines response. Combined PUFA and FA supplementation reduced infarct size, apoptosis, inflammation, neurotransmitter imbalance, and electrophysiological dysfunction in neonatal HI. The combination improved OPS recovery, reduced HIP incidence, and enhanced cognition and motor performance. It also suppressed proinflammatory cytokines and apoptosis markers, possibly via NF-κB pathway inhibition, while restoring memory and learning.

An experimental limitation of this study is the reliance on the MWM test as the primary measure of cognitive performance. Future studies incorporating additional assays may more clearly distinguish treatment-related cognitive effects from nonspecific motor improvements. Another limitation is the sensitivity of electrophysiological measures to technical variables, potentially confounding interpretations.

Future prospects of this study include the clinical translation of PUFA-FA therapy for neonatal HI management, exploring optimal dosages, long-term neurodevelopmental outcomes, and synergistic potential with other neuroprotective interventions.

ijpr-24-1-163943-s001.pdf

## Data Availability

All data generated or analyzed during this study are included in this published article.
