# Supplementary material for: Combined Omega-3 Fatty Acid and Folic Acid Supplementation Reduces Neonatal Hypoxic-Ischemic Brain Injury via Anti-inflammatory and Anti-apoptotic Mechanisms
Source: Iran J Pharm Res. 2025 Oct 24;24(1):e163943. doi: 10.5812/ijpr-163943 (PMC12606872; doi:10.5812/ijpr-163943)
Supplement: ijpr-24-1-163943-s001.pdf [file ijpr-24-1-163943-s001.pdf]

Appendix 1: Effects of PUFA and FA on HI-induced inflammation and apoptosis-related proteins. Representative Western blot for proteins from HI-induced tissues treated with PUFA and FA, respectively.

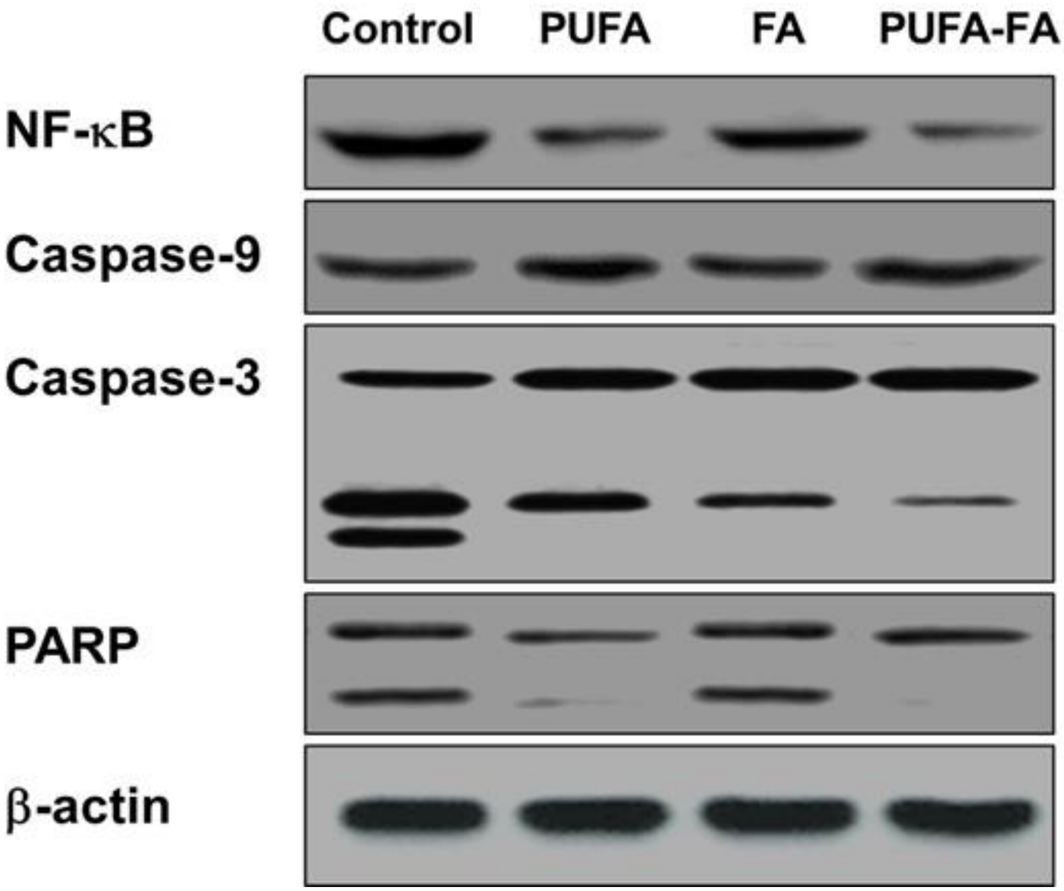

Appendix 2. The sequence of gene-specific primers used in the study.

**iNOS**	Forward 5’-GCAAACCCAAGGTCTACGTT-3’<br>Reverse 5’-GGAAAAGACTGCACCGAAGA-3’
**COX-2**	Forward 5’-CCTGTGCCTGATGATTGC-3’<br>Reverse 5’-CGGTGAAACTCTGGCTAG-3’
**TNFα**	Forward 5’-AGCACA GAAAGCATGATCCG-3’<br>Reverse 5’-CTGATGAGAGGGAGGCCATT-3’
**NF-κB**	Forward 5′-CCTCTGGCGAATGGCTTTAC-3′<br>Reverse 5′-GCTATGGAT ACTGCGGTCTGG-3′
**IL-1β**	Forward 5’-ACCTGCTGGTGTGTGACGTT-3’<br>Reverse 5’-TCGTTGCTTGGTTCTCCTTG-3’
**IL-6**	Forward 5’-GAGGATACCACTCCCAACAGACC-3’<br>Reverse 5’-AAGTGCATCATCGTTGTTCATACA-3’
**AIF**	Forward 5’-CCAGATATTTGAGCCACGCC-3’<br>Reverse 5’-ACCAGGCCTTAAGTAACCCC-3’
**BCL2**	Forward 5’-CACACACACACATTCAGGCA-3’<br>Reverse 5’-GGCAATTCCTGGTTCGGTTT-3’
**Caspase-3**	Forward 5’-AGGAGGGACGAACACGTCT-3’<br>Reverse 5’-CAAAGAAGGTTGCCCCAATCT-3’
**PARP**	Forward 5’-GCAGTCACCCATGTTCGATGG-3’<br>Reverse 5’-GCTTCTCTGGATCCACCATC-3’
**β-Actin**	Forward 5’-ATCACTATTGGCAACG-AGCG-3’<br>Reverse 5’-TCAGCAATGCCTGGGTACAT-3’
